# Bursts and Heavy Tails in Temporal and Sequential Dynamics of Foraging Decisions

**DOI:** 10.1371/journal.pcbi.1003759

**Published:** 2014-08-14

**Authors:** Kanghoon Jung, Hyeran Jang, Jerald D. Kralik, Jaeseung Jeong

**Affiliations:** 1 Department of Bio and Brain Engineering, Korea Advanced Institute of Science and Technology (KAIST), Daejeon, Korea; 2 Department of Psychological and Brain Sciences, Dartmouth College, Hanover, New Hampshire, United States of America; Imperial College London, United Kingdom

## Abstract

A fundamental understanding of behavior requires predicting *when* and *what* an individual will choose. However, the actual temporal and sequential dynamics of successive choices made among multiple alternatives remain unclear. In the current study, we tested the hypothesis that there is a general bursting property in both the timing and sequential patterns of foraging decisions. We conducted a foraging experiment in which rats chose among four different foods over a continuous two-week time period. Regarding *when* choices were made, we found bursts of rapidly occurring actions, separated by time-varying inactive periods, partially based on a circadian rhythm. Regarding *what* was chosen, we found sequential dynamics in affective choices characterized by two key features: (a) a highly biased choice distribution; and (b) preferential attachment, in which the animals were more likely to choose what they had previously chosen. To capture the temporal dynamics, we propose a dual-state model consisting of active and inactive states. We also introduce a satiation-attainment process for bursty activity, and a non-homogeneous Poisson process for longer inactivity between bursts. For the sequential dynamics, we propose a dual-control model consisting of goal-directed and habit systems, based on outcome valuation and choice history, respectively. This study provides insights into how the bursty nature of behavior emerges from the interaction of different underlying systems, leading to heavy tails in the distribution of behavior over time and choices.

## Author Summary

To understand spontaneous animal behavior, two key elements must be explained: *when* an action is made and *what* is chosen. Here, we conducted a foraging experiment in which rats chose among four different foods over a continuous two-week time period. With respect to *when*, we found bursts of rapidly occurring responses separated by long inactive periods. With respect to *what*, we found biased choice behavior toward the favorite items as well as repetitive behavior, reflecting goal-directed and habitual responding, respectively. We account for the *when* and *what* components with two distinct computational mechanisms, each composed of two processes: (a) active and inactive states for the temporal dynamics, and (b) goal-directed and habitual control for the sequential dynamics. This study provides behavioral and computational insights into the dynamical properties of decision-making that determine both when an animal will act and what the animal will choose. Our findings provide an integrated framework for describing the temporal and sequential structure of everyday choices among, for example, food, music, books, brands, web-browsing and social interaction.

## Introduction

Humans and non-human animals engage in a number of distinct activities on a daily basis, from working to attain resources to resting. Once engaged in a particular activity, such as foraging, they typically must select among multiple alternatives a number of times before they are satisfied. A systematic understanding of behavior, then, requires a characterization of the mechanisms that determine *when* to engage in an activity and to stop the activity, and *what* to choose, which includes choosing among multiple options multiple times. Although the processes governing *when* and *what* to choose have been studied in their own right, how both sets of underlying mechanisms together produce the dynamical properties of behavior over time remains poorly understood. To help characterize these fundamental mechanisms and their interaction, we examined and modeled the foraging decisions of rats in a paradigm designed to mirror the daily life of mammals composed of continuous free choices among multiple alternatives.

### Temporal dynamics: When to act

Rather than regular, like a metronome, or homogenous (i.e., a constant overall rate of activity), timing of behavior and/or events in humans, non-human animals, and natural phenomena is often non-homogeneous, with periods or *bursts* of high activity separated by long inactive periods [1], [2]. Examples in humans include e-mail [1], [3]–[7] or mail communication [8], library loans [3], financial trading [9], [10], on-line movie watching [11], internet browsing [3], [12], printing requests [13], and mobile communication [14], [15]; in non-human animals, locomotion [16]–[21], and flying patterns [22]; and in natural phenomena, rainfall [23], tsunamis [24], and earthquakes [2], [25]. A telltale diagnostic feature used to characterize non-homogeneous temporal processes is a *heavy tail* in the distribution of the inter-event intervals (i.e., the time interval between consecutive events) [1]. A heavy tail reflects a larger number of longer inter-event intervals than occurs with homogeneous Poisson processes (i.e., those in which the events occur at an overall constant rate, but are independent of one another).

Although a non-homogeneous process has been suggested as a universal feature of natural dynamical systems [2], different specific underlying mechanisms can lead to a heavy-tailed distribution of the inter-event intervals [26]. For example, it has been suggested that the bursty nature of human interactions results from the combined effects of different periodicities at different timescales: e.g., a circadian rhythm, as well as weekly, monthly, etc. cycles; and, in fact, bursty behavior can derive from a cascading non-homogeneous Poisson process model that combines multiple Poisson processes with different timescales [6], [27], [28]. At the same time, the bursty behavior of human interactions can also be induced by intrinsic correlations between actions [6], [27]–[31]. Indeed, bursty behavior might also derive from a combination of such processes, which we explore in the current study.

Here, we focus on foraging, a fundamental and frequent behavior for survival. Foraging mechanisms underlie the daily energy budget allocation across activities [32]–[34]. Unlike nature phenomena, feeding, and more generally, foraging behavior is influenced by both internal biological and external environmental factors: internal factors include preference, nutrition, memory, hunger and satiety; external factors include the daily light-dark cycle (leading to a circadian rhythm), seasonal and social/societal effects [32], [35]. Thus, the study of foraging behavior provides the opportunity to examine decision mechanisms that result from the interaction of important internal and external influences.

Feeding behavior has been studied in large data sets of farm animals, pets, and captive wild animals, including cattle, pigs, chickens, ducks, turkeys, rats, and dolphins [33], [35]–[39]. The temporal structure of feeding behavior consists of high frequency feeding events that are separated by relatively long non-feeding periods: *i.e.*, it is bursty [33], [38]. In the current study, our first objective was to test the hypothesis that foraging timing is based on bursty behavior that is influenced by both the level of satiety (internal) and by the daily light- dark cycle (external). Indeed, we found a heavy-tailed distribution of the inter-choice intervals (ICI, the time interval between two choices), reflecting a non-homogenous process. Moreover, the ICI distribution exhibited bimodality, reflecting distinctive processes for short and longer timescales: bursty behavior for short ICIs and circadian rhythmic activity for longer ICIs. To explain this bimodality in foraging behavior, we propose a dual-state model consisting of active and inactive states, with correlated behavior producing bursty activity in the active state, and relatively uncorrelated behavior influenced by a circadian rhythm in the inactive state.

### Sequential dynamics: What to choose

Once activity timing is characterized, the decision dynamics of which option to select and whether to continue selecting it over repeated choices must be specified [40]–[43]. Although progress has been made on characterizing outcome-driven behavior as governed by the goal-directed system [44], [45], and stimulus-driven behavior as governed by the habit system [45]–[48], it nonetheless remains difficult to predict an individual's preference and choice responses over a long period of time. For example, an individual's preference for different foods or music seems to fluctuate over time even when they have experienced the available options extensively and thus know all options well: e.g., even if one's favorite food is a hamburger, it typically is not eaten every single day. Thus, the underlying mechanisms that lead to dynamically changing preference-based choice behavior remains unclear, especially with qualitatively different rewards in stable environments, in which an agent ‘knows’ the reward contingencies and thus does not require further learning.

Therefore, the second objective of the current study was to help specify the mechanisms underlying seemingly unpredictable preference-based choices with (a) multiple qualitatively different options; and (b) repeated choices over an extended period in a stable environment that reflects real-world choice behavior. Here we extracted two distinctive features from an individual's dynamic choice sequence: (1) preference bias (i.e., the skew of the choice distribution based on the individual's rank order of choice options), and (2) choice persistence (i.e., the degree to which choices are repeated), which capture distinct underlying control processes that determine what to choose and whether to continue choosing it, respectively.

We found individual differences in preferences that nonetheless could be characterized by choice option rank, reflecting a value-based process, as well as some persistent choice behavior, in which choices tended to be repeated, with an increasing likelihood of repeating a choice as a run of identical choices increased, reflecting a preferential-attachment process. We then developed a dual-control model incorporating a combination of goal-directed and habitual control to describe the dynamical patterns of the choice sequences.

## Results

### Static description of choice behavior

We investigated the continuous choice behavior of 12 rats over the course of two weeks using a four-armed bandit task with four differently flavored pellets: chocolate, banana, coffee, and cinnamon. Each rat lived in an operant chamber for the entire two-week duration as a “closed economy” [49] with continuous access to water and the food pellets in the environment. Each trial was initiated by nose-poking in a lighted opening, after which four levers would extend from the opposite wall of the chamber (Figure S1). The rat then obtained one of the flavored pellets by pressing the corresponding lever.

**Figure 1:**
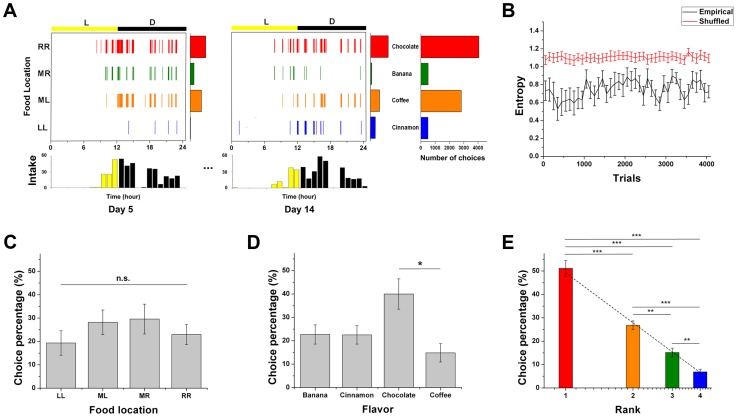
An example of the empirical choice patterns and the mean choice percentage of consumed pellets by food location, flavor, and rank. (A) The foraging behavior of a representative rat for two days, day 5 and day 14, illustrating the choice dynamics. The ordinate represents each food location/type and the abscissa represents the hour of the day. The light and dark cycles are denoted as yellow and black bars above each day's choice plot, with overall choice plotted per hour below the choice plot. The histogram to the right shows the total choices for the entire experiment. For subject 2, the rank 1 flavor (red color) was chocolate, located at the far right [RR]; the rank 2 (orange color) was coffee, middle left [ML]; the rank 3 (green color) was banana, middle right [MR]; finally, the rank 4 (blue color) was cinnamon, at the far left [LL]. (B) Entropy changes of representative data over trials. Black and red solid lines represent the entropy changes of the empirical and randomly shuffled data, respectively. (C) Mean choice percentage for specific food locations (LL, ML, MR, and RR) across subjects. (D) Mean choice percentage by flavor across subjects. (E) The mean choice percentage across subjects for each rank is shown in a log-linear scale. Choice percentage linearly decreases as a function of log(rank order). The dotted line is the log-linear fit (the slope  = −70.7±4.95 [mean ± s.e.m], adj. *R^2^* = 0.994). For all figures, error bars are standard errors of the mean (s.e.m). In C, D and E, a Dunnett-T3 post hoc test was conducted: *p<0.05, **p<0.01, ***p<0.001.

To examine *when* and *what* the animals chose, timing and choice sequences of lever-pressing activity for all rats were recorded for the entire experiment. With respect to *when* they chose, the animals actively foraged during the dark cycle and sporadically so during the light cycle as shown in Figure 1A. With respect to *what* they chose, we found dynamic changes in the animals' food choices, indicating that the rats did not commit themselves to a specific option but rather intermittently explored alternatives.

To assess the degree of the animals' exploration or exploitation, we first computed entropy of choice sequences every hundred trials [50], which is a measure of the uncertainty in choices, with zero being deterministic and solely exploitative and high entropy indicating a high degree of exploration (Figure 1B). We found that the entropy of choice sequences fluctuated to some degree throughout the experimental period. Although entropy changes varied slightly across subjects, overall, there was no significant tendency of entropy to decrease at the group level, indicating that the animals maintained some level of exploring alternatives throughout the experiment rather than converging toward a particular option. Next, we compared the entropy of empirical choice sequences with randomly shuffled ones, which removes any dependency on past choices, to determine whether the degree of exploration or exploitation depended on previous choice history (Figure 1B). We found that the levels of entropy in the empirical choice sequences were significantly lower than in randomly shuffled ones for all subjects (paired t-test, *p*<0.001). Thus, this result shows that previous choices influenced the current choice, consistent with other reports [40], [42], [51]–[54].

We next examined the amount of consumed pellets with respect to flavor, location, and rank. Rank was defined as the order of overall consumption of each food type for an individual, which would reflect the order of an individual's subjective values for the qualitatively different rewards. The percentages of mean choice for the four different locations – left (LL), middle left (ML), middle right (MR) and right (RR) – were not significantly different (one-way ANOVA, *F*(3, 44)  = 0.781, *p* = 0.511) (Figure 1C), reflecting the counterbalancing of flavor and position across subjects, and demonstrating that there was no preferred location overall. In addition, to test whether there were differences in effort to reach each lever location from the initial nose poke position, we compared the response latencies between nose-poke and lever pressing for each location. The response latency medians across locations were not significantly different (one-way ANOVA, *F*(3, 44)  = 0.009, *p* = 0.998), suggesting that the animals' response vigor for each location was similar [55].

The consumption rates for each flavor were significantly different (one-way ANOVA, *F*(3, 44)  = 5.043, *p*<0.01): the chocolate flavor was statistically more consumed than the coffee flavor at the group level (Dunnett-T3 post hoc test, *p* = 0.021) (Figure 1D), although this was not the case for all subjects (e.g., Figure 1A); nonetheless, all rats showed distinct individual preferences among the different flavors.

Since the rats exhibited individual differences in preference, and since quality has no obvious natural corresponding number to represent its value (especially when quality was essentially flavor), we analyzed choice behavior based on rank, which should be driven by an individual's subjective values of the options, and which provides a common scale to compare individuals. Comparing the percentages of mean choice for rank, there was a clear difference between food pellets of different ranks as shown in Figure 1E (one-way ANOVA, *F*(3, 44)  = 74.897, *p*<0.001; Dunnett-T3 *post hoc* test).

Interestingly, choice rate appeared to decrease by nearly half as rank increased. To confirm this tendency, we transformed the percentage of food choice by rank to a log-linear scale. We found that the mean distribution of the choice percentage *p* as a function of rank *r* was well described by the log-linear distribution (Figure 1E), where the slope of *p* versus log(*r*) was −70.7±4.95 (mean ± standard error of the mean [s.e.m.], adj. *R^2^* = 0.994), indicating that preference was highly skewed toward the higher rank.

### Temporal features of choice behavior

**Figure 2:**
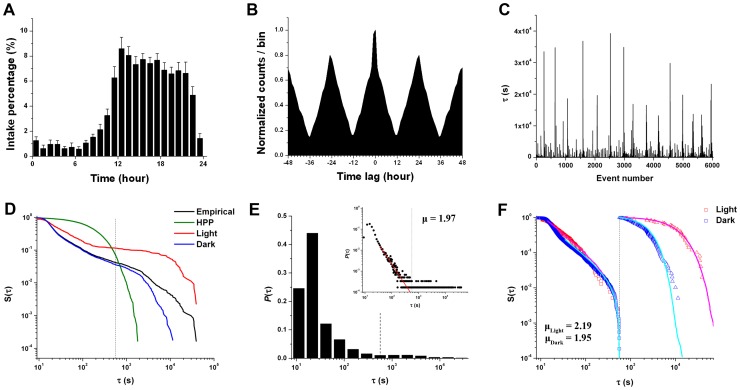
Temporal features of the foraging behavior. (A) The variation of intake percentage during a day averaged over all rats. (B) The autocorrelogram of the time series of foraging behavior over all rats. The period of the foraging behavior is measured by extracting the pitch of the average autocorrelogram. The time interval between peaks is 24 hours, which is consistent with the animals' circadian rhythm. (C) The inter-choice interval (ICI) sequence for an example rat (subject 4). Short ICIs are abundant while long ICIs are intermittently observed. (D–F) display example results for the same rat. (D) The cumulative distribution of ICIs longer than a given ICI is heavy-tailed in a log-log scale. The distribution of the empirical data (black solid line) is compared to what would be predicted from a homogeneous Poisson process (HPP) (green solid line). The red and blue solid lines denote the cumulative ICI distribution for the light and dark cycles, respectively. (E) The probability density function of the bimodal ICI distribution. The power-law fitted to the probability density function for short ICIs is shown in a log-log scale (the red line in the inset) (F) Separate cumulative ICI distributions for short and longer ICIs in the light (red) and dark (blue) cycles. Squares and triangles denote short and longer ICIs, respectively. For short ICIs, the magenta and cyan lines represent synthetic power-law distributions with the upper bound 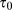 fitted to the empirical data for the light and dark cycles, respectively. For longer ICIs, the magenta and cyan lines represent synthetic Weibull distributions fitted to the empirical data for the light and dark cycles, respectively. (D–F) The black dotted line represents the time constant 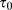, which separates events into independent bursts. All the exponents were obtained by maximum likelihood estimation (MLE).

Examining the timing characteristics of the choice behavior in more detail, we found periodic changes in food consumption. First, the animals consumed more pellets during the dark than the light cycle (Figure 2A). To investigate the relationship between the foraging pattern and the daily light-dark cycle (i.e., a potential circadian rhythm effect), we measured the periodicity of the foraging pattern by calculating the time interval between peaks in the average autocorrelogram. The rats' foraging pattern period was approximately 24 hours, consistent with their circadian rhythm (Figure 2B), indicating that it was one of the key factors that determined foraging timing in general. The remaining issue was how the *specific* timing of foraging was determined at a short timescale.

We characterized the underlying action dynamics by analyzing the features of the inter-choice interval (ICI) distribution. We found that the majority of ICIs were short, but very long ICIs also sporadically occurred, indicating that there were bursts of activity separated by relatively long inactive periods (Figure 2C). To measure this burstiness in the timing of foraging behavior, we used a burstiness index *B*, defined as 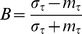, where 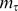 and 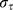 are the mean and the standard deviation of the ICI distribution, respectively [31]. *B* ranges between −1 and 1: *B* = 1 is the most bursty signal, *B* = 0 is neutral, and *B* = −1 is a completely periodic signal. We found that *B* of the foraging behavior was 0.794±0.008 (mean ± s.e.m), indicating that the majority of activity was densely concentrated in short durations.

Next, to characterize a memory effect, we calculated the correlation coefficient of consecutive inter-choice intervals, which is defined as 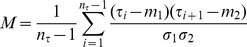, where 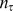 is the number of ICIs measured from the timestamps, and *m_1_* (*m_2_*) and 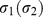 are the mean and standard deviations of the ICIs 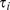
's (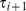
's), respectively [31]. *M* ranges between −1 and 1: *M* is positive when the length of the current ICI is positively proportional to the length of the previous ICI; whereas, *M* is negative when the length of the current ICI is inversely proportional to the length of the previous ICI; *M* = 0 is neutral; and *M* = −1 is a completely periodic signal. We found that *M* of the foraging behavior was 0.046±0.006 (mean ± s.e.m), indicating that the foraging activity had a relatively low correlation between consecutive ICIs.

The bursty nature of the foraging behavior was reflected in the heavy-tailed ICI distributions. The cumulative distribution of ICIs, which is the probability of ICIs longer than a given ICI (i.e., the survival function), exhibited a heavy tail that was clearly seen in a log-log scale, representing a deviation from an exponential distribution resulting from a simple homogeneous Poisson process (Figure 2D). This indicates that the time interval between spontaneous behaviors is not simply governed by a random process, but is modulated in a more sophisticated way by other processes at a longer timescale. In addition, heavy tails were also observed in the distributions of ICIs in both the light and dark cycles (Figure 2D).

Interestingly, the empirical ICI distribution exhibited bimodality (Figure 2E). For short ICIs, the probability density function of the ICIs was highly left-skewed; whereas for longer ICIs, the probability density function did not appear to reflect the same left-skewed characteristic. The highly left-skewed component of the distribution for short ICIs was well fit by the power-law (*p* = 0.68±0.09 for the fit to the power-law distribution—i.e., the empirical and power-law distributions were not significantly different; see “Estimation of parameters in the inter-choice interval (ICI) distribution” in Material and Methods) (Figure 2E inset). The second component of the distribution for longer ICIs appeared to follow the Weibull distribution, exhibiting a stretched exponential decay; however, with combined light and dark cycles, the empirical and Weibull distributions were significantly different. When we decomposed the overall ICI distribution into the component light and dark cycles, however, the distributions of the short ICIs for both cycles followed the power-law distribution, and the distributions of the longer ICIs for both cycles followed the Weibull distribution (Table 1 and Figure 2F).

**Table 1:** Parameter estimates of the bimodal ICI distributions.

	Overall	Light	Dark
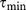	39.92±2.41	40.32±2.45	39.92±2.41
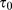	540.47±55.60	540.47±55.60	540.47±55.60
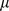	2.09±0.05	2.21±0.07	2.07±0.05
*p-value* (power-law)	0.68±0.09	0.50±0.09	0.45±0.07
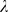	(4.16±0.32) ×10^3^	(1.20±0.15) ×10^4^	(2.74±0.20) ×10^3^
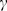	0.91±0.02	1.15±0.07	1.23±0.06
*p-value* (Weibull)	0.03±0.01	0.85±0.05	0.15±0.05

The estimated parameters of the bimodal ICI distributions from 12 subjects. Values are given as mean (s.e.m). Overall: Overall ICI distribution; Light: ICI distribution in the light cycle; Dark: ICI distribution in the dark cycle. See text for parameter definitions.

Thus, the cumulative bimodal ICI distributions for both the light and dark cycles could be described as the following:
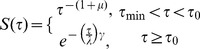
where 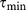 is the lowest time boundary, 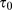 is a time constant used to separate activities into independent bursts, µ is the power-law exponent, *λ* is a scale parameter, and *γ* is the shape parameter of the distribution. We calculated the value of *τ_0_* as the local minimum of the bimodal distribution of ICIs, which separated the short and longer ICIs in the distributions. The estimated parameters of the bimodal ICI distributions are shown in Table 1 (see “Estimation of parameters in the inter-choice interval (ICI) distribution” in Material and Methods for details). This bimodality in the ICI distributions suggests (a) different underlying processes at different timescales of ICIs, and (b) similar underlying processes in both the light and dark cycles leading to the power-law and Weibull distributions. We take up these implications in the discussion.

When comparing the fitted parameters in the light and dark cycles, we found that the distributions for longer ICIs between the light and dark cycles exhibited different exponential decays reflected in the scale parameter *λ* (light: [1.20±0.15] ×10^4^, dark: [2.74±0.2] ×10^3^, Sign test, *p*<0.001), whereas the power-law distributions for the short ICIs in both cycles appeared to have similar slopes (light: 2.21±0.07, dark: 2.07±0.05, Sign test, *p* = 0.146) (Table 1 and Figure 2F). This finding comparing the light and dark cycles implies that the underlying mechanism governing longer ICIs was influenced by the circadian rhythm; whereas, the mechanism governing short ICIs may have been more weakly influenced by the circadian rhythm.

### Sequential features of choice behavior

**Figure 3:**
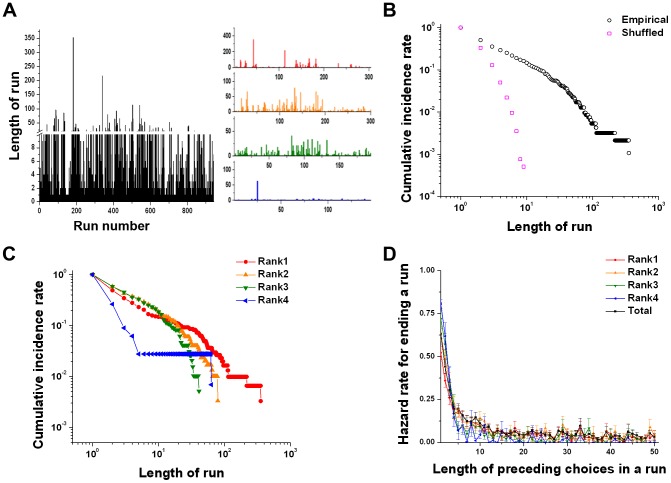
Sequential features of the empirical choice patterns. (A) A trial-dependent change of run lengths for one example rat is shown both for all runs together and separated by rank. Short runs are frequent while a few long runs are intermittently observed. (B) Cumulative distribution of runs longer than a given length of run in a log-log scale for one example rat (subject 5). The cumulative run distribution of the empirical data compared to randomly shuffled data with no trial-by-trial dependencies. (C) Cumulative run distribution of each rank for the same rat (subject 5). (D) The hazard rate for ending a run with respect to the number of preceding choices in a run averaged over all rats.

We next analyzed the choice patterns to examine the sequential dynamics governing *what* is chosen over trials. First, we determined how long the rats continued to make the same choice. We defined a “run” as a series of consecutive identical choices. A trial-dependent change in a distribution of runs was then calculated, as shown in Figure 3A. The cumulative distribution of runs, defined as the probability of runs longer than a given length of run (i.e., the survival function), revealed a heavy tail in a log-log scale (Figure 3B), indicating that the choice pattern consisted of a large number of short runs and a few extremely long runs.

To test for a sequential dependency of previous choices, we compared the run distributions of the empirical sequences with those of randomly shuffled sequences of the same data for each rat. The randomly shuffled sequence has no dependency on previous choices yet maintains the same choice frequency as the empirical data. The cumulative run distribution of the empirical data was significantly different from that of the randomly shuffled choice sequences for all subjects (Monte Carlo hypothesis testing, *p*<0.001) [6]. This result indicates that the choice sequences were highly influenced by the choice histories [40], [42], [52], [54].

In addition, we examined whether there was an effect of choice history regardless of rank by comparing the run distribution of empirical data for each rank with that of randomly shuffled data (Figure 3C). Although the lower ranking flavors had fewer long runs than the higher ranking ones, the run distribution of the empirical data for all ranks was significantly different from those of the randomly shuffled choice sequences for all subjects, with the exception of the fourth rank for two of the twelve subjects (Monte Carlo hypothesis testing, *p*<0.001) [6]. The shared heavy-tailed feature of the run distribution for every rank suggests that the underlying processes determining whether a run would continue were relatively insensitive to reward outcome.

Conducting a simple calculation with the cumulative distribution of runs, we obtained the hazard rate for ending a run as a function of the number of preceding choices in a run for each rank, i.e., the conditional probability of ending a run at a given length of a run (Figure 3D). We found that the hazard rate for ending a run decreased logarithmically and converged relatively quickly to approximately zero in all ranks. This indicates that a run was more likely to be terminated when the length of the preceding choices in a run was short; and the run was more likely to continue when the length of the preceding choices in a run was increased. In addition, the hazard rate converging to zero resulted in extremely long runs regardless of rank; indeed, there was no significant difference in the decreasing rate of the hazard rate between ranks (one-way ANOVA, *F*(3, 44)  = 0.666, *p* = 0.577). Thus, in general, the rats were more likely to choose what they had chosen previously, irrespective of outcome, reflecting a status quo bias or *preferential-attachment process* that tends to continue a run until switching one's choice finally becomes more compelling.

## Models

We next propose two models that capture the temporal and sequential dynamics of free choice behavior. First, we account for the temporal features of the decision patterns by proposing a dual-state model that captures both the bursty property and the circadian rhythm influence on the rats' choice behavior, leading to a heavy-tailed distribution of the ICIs with bimodality. Second, we account for the sequential features of the decision patterns by proposing a dual-control model that incorporates the combination of two distinct control processes: goal-directed and habit control, which characterize the bias in choice frequency with respect to rank order and the heavy-tailed nature in the run distribution, respectively.

### Temporal dynamics model

**Figure 4:**
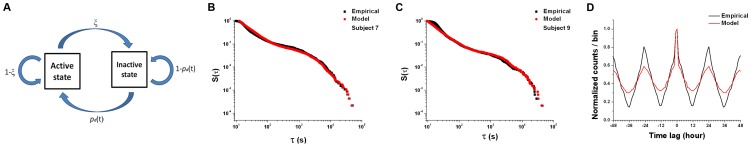
Comparison of the simulation of the dual-state model with the empirical data. (A) Schematic diagram for the dual-state model. (B–C) Cumulative ICI distributions of the empirical data (black squares) from two example rats and the simulated data from the dual-state model (red circles) in a log-log scale. (D) Autocorrelograms of the empirical and the simulated data averaged across rats. The black and red lines denote the empirical and the simulated data, respectively. The time interval between peaks of the simulated data is 24 hours, which is consistent with that of empirical data.

A bimodal distribution has been suggested as a mixture of distinct distributions formed by different underlying processes [14], [25], [38]. We found that the empirical ICI distribution underlying the foraging behavior under free conditions exhibited bimodality with the power-law and Weibull distributions for short ICIs and longer ICIs, respectively. To characterize the bimodal temporal dynamics, we propose a dual-state model that can provide an integrative account of both the bursty and periodic features of the foraging behavior. The model consists of an active state and an inactive state, which executes correlated actions in bursts in the active state, and elicits intermittent uncorrelated actions in the inactive state (Figure 4A).

We consider an animal to be in an active state when the animal exhibits a high frequency of activity, with short ICIs that are less than a certain time period 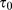, and we assume that the events within the active state are correlated due to the influence of the motivational drive [2]. In our case, the motivational drive for feeding is to appease hunger (i.e., reach satiation). A known physiological mechanism underlying short-term regulation of feeding (within a meal) is that feeding is governed by a feedback mechanism from the delayed gastrointestinal aftereffects of eating [36]; the digestion of food inhibits eating, but the inhibitory effect is delayed. Here, we focus on the delay between the swallowing of food and the digestion of food, resulting in the delayed satiety signal as feedback. And this characteristic of feeding leads us to propose a *satiation-attainment process*, i.e., an active waiting process based on feedback for upcoming satiation within each active state. In this process for the active state, we assume that whenever animals eat, they wait for the feedback signal by which they determine whether to eat more or stop. In other words, animals initiate eating and wait until they receive the satiety signal, which informs them that satiation is attained. If the satiety signal is lower than the satiation threshold, they would continue to eat and wait for the next feedback signal. Thus, the waiting time between eating and the feedback signal is important to determine time intervals between actions in an active state. Instead of a constant time delay of feedback, we assume that there is a non-linear relationship in the waiting time between eating and the feedback signal. A number of studies on human dynamics have suggested that the waiting time based on feedback in human communication patterns follows a power-law distribution [1], [7], [8], [14]. Considering a similarity in the waiting process for feedback between feeding and human communication, we assume that the waiting time between eating and the feedback signal follows a power-law distribution; in active states, the probability density function of the time interval between choices is 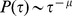 for 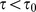 where 1<µ<3.

In addition, an animal is considered to be in an inactive state when there is a period of inactivity longer than 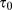; and thus the inactive state is defined as the time between the last event in a given active state and the first event in the next active state, which by definition, is longer than 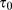. We model timing in the inactive period with a non-homogeneous Poisson process with the inactivity rate 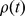, i.e., the reciprocal of the mean inactive duration as a function of time. To capture the strong influence of the circadian rhythm on the longer ICIs, two temporal properties of the inactivity rate are further specified. First, the inactivity rate 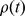 depends on time in a periodic manner, as expressed by the equation 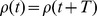, where *T* is the period of the process. Since the animals' periodic activity is modulated by a circadian rhythm, we set the period *T* as 1 day. Second, the inactivity rate 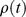 is proportional to the daily distribution of choice behavior in the inactive state, 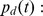 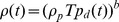, where 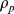 is the average rate in the inactive period, 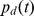 is the probability of beginning an active state during a particular hour of the day 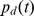 [6], and *b* is the shape parameter. To quantify the transition between active and inactive states, we assume that a state transits from the active state to the inactive state with a probability ξ after each choice and remains in the active state with probability 1 – ξ.

### Sequential dynamics model

With the computational processes that determine *when* choices are made specified, we next delineate those that determine *what* choices are made. Here, we propose a simple heuristic model that accounts for two key sequential features of decision-making: (1) the heavy-tailed nature of the run distribution, reflecting choice persistence as habitual behavior, and (2) the biased rank distribution, reflecting goal-directed outcome valuation.

First, to account for persistence in choice behavior, we assume an underlying preferential-attachment process, which has been proposed as the mechanism underlying heavy-tailed distributions [42], [56], [57]. In this process, the probability of continuing a run increases as a run proceeds (thus, it also has been called the “rich get richer” process). We suggest that the same mechanism underlies choice behavior, in which the probability of choosing a particular option is proportional to the number of times the option was chosen previously. The process may underlie response persistence found in choice behavior in humans and nonhuman primates [40], [42], [58], [59]. In addition, the preferential-attachment process occurs regardless of outcome type, reflecting its property of insensitivity to outcome, which is a defining feature of habitual behavior (Figure 3D). Thus, this process may underlie the acquisition and maintenance of habits. We therefore more generally call this mechanism, *habitual control*.

In the habit system, in addition to the preferential-attachment process, we apply a leaky integrator to the dynamic trial-by-trial model of habitual behavior, in which the integrated choice frequency over previous trials is discounted as a function of the distance passed from a given trial [52], [57], [60], [61]. Thus, this integrator includes the effect of past choices [42]. Because the preferential-attachment process is insensitive to outcome, we assume that the discount rate is identical for all options regardless of rank. In habitual control, the action value of a particular option *i* at trial *t*, 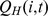, is determined by the local choice history of that option with leakage: 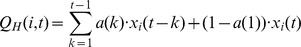
where 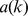 is a weighting coefficient for choices occurring 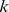 trials ago with an exponential decreasing profile, equal to 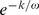, where 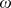 is a free parameter for the decay constant, and 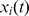 is a binary vector denoting a chosen option *i* on trial *t*. The choice vector 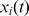 is 1 if option *i* was chosen on trial *t* and 0 if the option was not chosen on that trial.

Second, for goal-directed control, we use a temporal difference (TD) reinforcement learning algorithm that updates the action-value on each trial according to its prediction error [62]–[66]. The TD learning algorithm provides a theoretical framework for instrumental reward learning in which actions must be chosen to optimize long-term rewards [63], [67]. In addition, we incorporate a decay factor, which updates the chosen option and decays unchosen options [66], [68], [69]. Thus, at each trial *t*, the action value for the chosen option *c* and for the unchosen option *u* are updated according to:
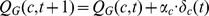


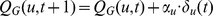
where 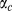 and 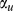 are learning rates and 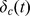 and 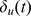 are the reward prediction errors at given trial *t* for the chosen and unchosen options, respectively. The reward prediction errors, i.e., the difference between the expected and received reward values, for the chosen and unchosen options are as follows:



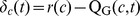


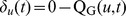
where 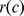 is the reward value for the chosen option. We deductively estimated the reward value based on the mean choice rate across days, *R*: 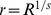 where 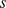 is a parameter of sensitivity of behavior to differences in reward values among alternatives [70]. We refer to this outcome-driven process as “goal-directed.” The goal-directed process plays an important role in determining the initial choice for a new run on the basis of value, which in turn generates a certain degree of bias toward a more valued option.

Finally, for action selection, to capture the effects of both the habit and goal-directed systems on choice behavior, the goal-directed value 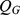 and habit value 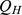 are derived in parallel [71]. We then assume that the probability to choose an option *i* at trial *t*, 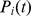, is determined according to a softmax choice function [63]:
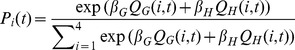
where the softmax inverse temperature parameters 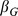 and 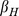 represent the degree to which a choice is focused on the highest-valued option in goal-directed value 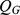 and habit value 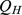, respectively. Note that, together, the combination of goal-directed and habit systems create two key features of sequential dynamics: a bias among choice options and a bursting property in which very long runs are interspersed among a majority of short runs.

### Modeling results

#### Temporal features of the model

We conducted simulations based on the dual-state model to examine how well the model captured the temporal dynamics of foraging behavior (Figure 4A). We set a time constant 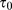 as a free parameter, which separates the choice sequences into independent bursts, and we identified the active and inactive states based on the constant. We then estimated the average rate in the inactive period 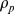, the transition probability from the active and passive states 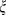, and the probability of the active state occurring at a particular time of day 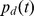 from the empirical dataset (see “Estimation of parameters in the inter-choice interval (ICI) distribution” in Material and Methods for details) (Table 2). Using these parameters and free parameters for the power-law exponent 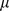, the lowest time boundary 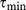, and the shape parameters for light and dark cycles, 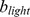 and 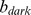, we generated simulated foraging time series for each rat (Table 2).

**Table 2:** Parameter estimates of the dual-state temporal model.

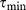	10.2±0.42
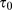	532.0±54.4
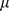	2.01±0.01
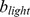	1.01±0.005
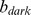	1.03±0.003
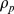	(2.43±0.21) ×10^−4^
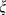	(5.34±0.87) ×10^−2^

The estimated parameters averaged over 12 subjects for the dual-state temporal model. Values are given as mean (s.e.m). See text for parameter definitions. See text for parameter definitions.

First, we compared the cumulative ICI distributions of the empirical data with those of simulated data. The cumulative ICI distributions of empirical and simulated data were similar to each other, with the simulated data exhibiting a heavy tail with bimodality in agreement with that of the empirical data (Figure 4B–C). Next, we compared the periodicity of the simulated and empirical data. The simulations exhibited the 24-hour period consistent with a circadian rhythm in the empirical data (Figure 4D).

#### Sequential features of the model

To determine whether the dual-control choice model could capture the two key sequential features in the choice patterns – a biased rank distribution and a heavy-tailed run distribution – we simulated choice sequences for individual rats with the best-estimated free parameters and then compared the fits of the models to the empirical data. We estimated the free parameters of the model for each rat by minimizing the negative log-likelihood of the individual choice sequences (Table 3) [72].

**Table 3:** Parameter estimates of the dual-control choice model.

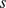	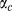	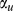	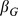	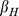	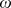
8.08±1.14	0.27±0.03	0.16±0.04	3.57±0.16	1.34±0.18	73.4±22.9

The estimated parameters from 12 subjects for the dual-control choice model. Values are given as mean (s.e.m). See text for parameter definitions.

We found that the predictions of the dual-control model significantly deviated from that of a random choice model for all subjects, indicating that the model fit to the empirical data was significantly better than chance (the pseudo- *r* ^2^ results in the far right column of Table 4). The nested models of the dual-control model (goal-directed and habitual control alone) and their variants also showed a significant deviation from the random choice model (Table 4), indicating that each component of the dual-control model alone also fit the data significantly better than chance.

**Table 4:** Comparisons among choice models.

	-LL	LRT	Number favoring Dual	BIC	p- *r* ^2^
Dual (Goal_c+u_+Habit)	3701±397	-	-	7454±794	0.51±0.05
Goal_c+u_	3716±396	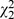 = 30.5	10/12	7467±792	0.51±0.05
		*p*<2.40e-7			
Goal_c_	5952±316	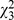 = 4501.7	12/12	11930±632	0.23±0.03
		*p* = 0			
Habit	4002±423	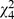 = 601.7	12/12	8021±847	0.47±0.05
		*p* = 0			
Goal_c_+Habit	3858±403	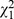 = 312.8	12/12	7758±806	0.49±0.05
		*p* = 0			

Qualities of behavioral fits of choice models. Values are given as mean (s.e.m). –LL, Negative log-likelihood; LRT, Likelihood ratio test statistic against the dual-control choice model (Dual); BIC, Bayesian information criterion; p-*r* ^2^, pseudo-*r* ^2^ statistic.

**Figure 5:**
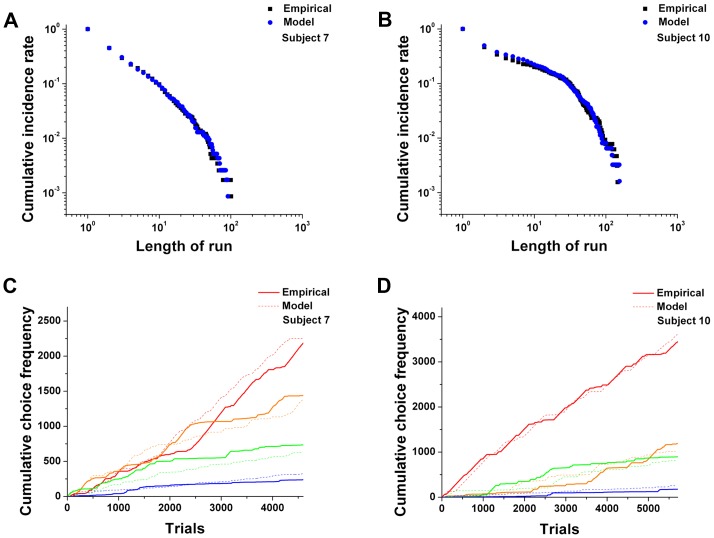
Comparison of a choice sequence generated from the dual-control model with the empirical data from two representative rats. (A–B) Cumulative run distributions of the empirical data for the two representative rats and the simulated data in a log-log scale. The black squares denote the empirical data and the blue circles the simulated data. (C–D) Cumulative choice frequency graphs for each rank for both the empirical data (solid lines) and simulation (dashed lines). Red, orange, green, and blue represent the rank order from rank 1 to rank 4, respectively.

Figures 5A and B show two examples of the close agreement between the cumulative run distributions of the empirical data and the simulated data generated by the dual-control model. The simulations of the dual-control model exhibited a similar degree of bias in the rank distribution as seen in the empirical data (see “Choice model comparison” in Text S1 for details). In addition, the cumulative choice frequency graph of both the empirical and the simulated data evolved similarly across trials (Figures 5C and D), indicating that the model captured the dynamic changes in choice behavior.

Finally, to test whether the dual-control model provided a better fit to the empirical choice behavior than its nested models, we conducted comparisons among models: (1) The goal-directed control model alone with update for the chosen option and decay for the unchosen options (Goal_c+u_); (2) the goal-directed control model alone with update for the chosen option only, i.e., standard TD learning for the chosen option (Goal_c_); (3) the habitual control model alone (Habit); (4) the submodel of dual-control (Goal_c_+Habit) composed of the mixture of Goal_c_ (with update for the chosen option only) and Habit; and (5) The dual-control model (Dual) composed of the mixture of Goal_c+u_ (with update for the chosen option and decay for the unchosen options)and Habit (see “Choice model comparison” in Text S1 for details). The dual-control model was superior to the Goal_c+u_ model in 10 out of 12 subjects and to the other submodels in all subjects according to the Bayesian information criterion (Table 4) [73].

## Discussion

In this study, we examined how dynamic foraging behavior can arise even in a stable, certain, and over-trained environment. Specifically, we uncovered underlying structures of the *when* and *what* components of foraging behavior and accounted for these components with distinct computational mechanisms.

### Temporal features of choice dynamics

Regarding *when* choices were made, we found bursts of rapidly occurring actions separated by time-varying inactive periods, partially based on a circadian rhythm. These characteristics of foraging behavior were reflected in a bimodal inter-choice interval (ICI) distribution comprised of a power-law for the short timescale (i.e., short ICIs) and the Weibull distribution for the longer timescale (i.e., longer ICIs). Although the specific mechanisms of the bimodal inter-event times could vary across different systems [9], [10], [14], [24], [25], [74], [75], a common dynamical feature of the underlying mechanisms appears to be the combination of distinct processes at different timescales [14], [25], [37]. To capture the temporal dynamics underlying foraging behavior, we propose a dual-state model consisting of active and inactive states for short and longer timescales based on a satiation-attainment process for bursty activity in the active states, and a non-homogeneous Poisson process for longer inactivity between bursts in the inactive states.

For the short timescale, we found an inverse square power-law distribution for short ICIs with exponent 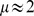. Interestingly, a recent study in human short message correspondence, which requires feedback between individuals, suggests that the waiting time of the bursty communication follows the power-law distribution with exponent 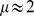. Analogously, a satiation-attainment process could govern the timing of feeding activity by waiting for satiation feedback. In fact, it is well known that short-term feeding is regulated by feedback from the delayed gastrointestinal aftereffects of eating and satiety signals: based on this feedback, meal termination is determined [36], [76].

For the longer timescale, we found that longer ICIs follow the Weibull distribution in both the light and dark cycles. At the same time, the cumulative distributions of the longer ICIs in the light and dark cycles exhibited different decay rates. One possible account for this difference between the light and dark cycles is the effects of the circadian rhythm on the motivation for general activity [77], as well as on specific activities such as sleep and feeding. A previous study on sleep-wake transitions suggested that long and periodic awake episodes in the sleep period are governed by the homeostatic sleep drive [78]. Thus, the long inactivity patterns might result from sleep-wake patterns. However, in contrast to the previous study, we found long inactivity patterns not only in the light cycles (the sleep period in rats) but also in the dark cycles. Thus, although it appears that sleep-wake patterns can contribute to generating longer inactivity patterns in the sleep period, it does not appear that the long inactivity patterns in the current study can be explained entirely by the homeostatic sleep drive.

The longer ICIs are likely influenced by the homeostatic hunger drive. The Weibull distribution is commonly used to describe the time to a first event [79], which in our case would be the time to the next foraging bout, i.e., to the next burst. Consistent with the use of the Weibull distribution, a threshold mechanism can be implemented in controlling the timing between independent bursts [36]. Physiological regulatory mechanisms associated with satiety have been suggested to control the time interval between bouts in a wide range of animals: when the satiety signal reaches or rises above a certain threshold, animals stop eating; whereas, when the satiety signal falls below the threshold due to a long period of non-feeding, they initiate eating again [35], [36], [38]. In fact, a simple “bang-bang” control system has been proposed that describes such a straightforward mechanism that uses the comparison of a satiety signal to a threshold, with the first ‘bang’ occurring when below threshold, and the other once threshold is reached. Moreover, a change in the threshold level between night and day (and potentially from hour-to-hour) provides a possible time-varying mechanism for the time interval between meals [36].

### Sequential features of choice dynamics

Regarding *what* was chosen, we examined sequential dynamics underlying free choice patterns in a stable environment in which an animal could obtain the food items with certainty. Despite the certainty of reward delivery as well as a stable reward value, the rats exhibited rich choice dynamics rather than a monotonous pattern. In contrast to the popular notion that goal-directed behavior gives way to automatic habitual behavior in a stable environment [51], we found that the entropy of the choice patterns remained relatively stable over the course of the experiment, suggesting that the animals maintained a balance between exploration and exploitation. This sustained balance suggests that the goal-directed process is indeed maintained, in order to maximize rewards even in stable, deterministic environments [80]–[82]. Instead of persisting with a particular option as a habit, maintaining the balance allows animals to monitor the environment for potential changes and to adapt more flexibly if and when changes occur. Such rich choice dynamics reveal that internal factors such as the value of available options and the previous choice history [42], [55], [83]–[85] play a critical role in generating choices.

To extend beyond quantity-based decision-making, in this study we focused on the dynamics underlying choices based on individual preference with respect to qualitatively different rewards with different flavors. Because qualitatively different rewards have no obvious corresponding numerical value, we used *rank* as a means to measure their relative subjective value based on individual preference. Indeed, we found a highly biased rank distribution toward an individual's favorite option. This rank distribution reflects one of goal-directed behavior's key properties, that action selection is guided by the value of outcomes to the individual [44], [45], [86], [87].

In our dual-control model, the subjective value of qualitatively different rewards was deductively estimated from each individual's choice behavior on the basis of the generalized matching law in which the choice rate matches the relative value of the options modulated by a sensitivity parameter [43], [52], [57], [70], [81]. When we tested the model with the empirical data, the reward value estimation resulted in small differences between the options. At the same time, the goal-directed control process successfully captured the highly biased rank distributions. This suggests that quality-based choice behavior can be modeled by a value-based process, and that a small difference in subjective values for quality can nonetheless generate large differences in choice behavior by an internal amplifying control process.

To capture both the value-based and internal amplifying control processes, we modified the standard TD algorithm [63] to update the action value for the chosen option according to the outcome, and at the same time, to apply a decay to the unchosen options [66], [68], [69], [88]–[90]. Thus, internal value representations for all available options are updated in this model. This process provided a superior fit to the empirical data. The addition of value updating of all options to the standard TD algorithm results in the action value of the chosen option increasing over trials and that of the unchosen options decreasing. The decay of action values for the unchosen options in turn results in a larger reward prediction error when the unchosen option is later chosen. Thus, the decay effect can lead to dynamic changes in choices due to variation in reward prediction errors over trials even in a stable environment.

For habitual control, the dynamic choice patterns revealed two key characteristics of habitual behavior: repeated responses and insensitivity to outcome [44]–[46]. We found that the rats intermittently generated very long runs throughout the experiment, resulting in a heavy tail in the run distribution. Furthermore, the run distributions for all ranks exhibited this heavy-tailed property, indicating a general persistence or ‘stickiness’ to past choices regardless of outcome. This insensitivity is consistent with a recent study on monkeys showing heavy-tailed run distributions regardless of reward types (water and apple juice) [58], as well as other studies showing that trial-by-trial choice dynamics are strongly influenced by past choices [40], [42], [54], [57]. While a large number of studies that model goal-directed and habitual processes have recognized this effect of previous choices on current ones [40], [54], [59], [91]–[94], the detailed process underlying choice persistence has not been fully described. We have built upon this work by delineating the mechanism more explicitly.

## Conclusions

Our empirical study shows that even in stable environments animals can exhibit rich temporal and sequential behavioral dynamics. In addition, our modeling work demonstrates how the interaction of different underlying processes can give rise to dynamic activity patterns. A dual-state model suggests that dynamic transitions between active and inactive states produce bursty and circadian rhythmic properties of temporal dynamics. A dual-control model suggests that goal-directed and habitual control processes cooperate, rather than compete, to generate sequential dynamics of choices that lead to a better option and increase the reliability of a performed action. Considering the ubiquity of decision-making in the lives of animals and in our everyday lives, temporal and sequential dynamics of spontaneous choice behavior raise the intriguing possibility that such dynamics derive from a harmonious collaboration of multiple underlying neural control systems – a collaboration that, when discordant, may lead to aberrant decisions such as binge eating or other forms of addictive behavior.

## Materials and Methods

### Ethics statement

All procedures of animal care and experiment were performed according the KAIST guidelines for the care and use of laboratory animals and approved by the KAIST Institutional Animal Care and Use Committee.

### Subjects

Twelve eight-week-old naïve male Sprague Dawley rats weighing 250–350 g were used in the study. The rats had all experienced a standard laboratory diet, and none had experience with the flavors used in the experiment.

### Behavioral testing

Each rat was individually housed in an operant chamber (see Text S1 for details and Figure S1) and maintained on a 12-h light/dark cycle for two weeks. The animals had *ad libitum* access to water. Food was available according to the experimental task described below. The four types of flavored 45 mg pellets—chocolate, banana, coffee, and cinnamon—were made from the same meal substrate (Bio-Serv, Frenchtown, NJ, USA) and were consequently matched with regards to all macro- and micro-nutrients. The locations of the flavored pellets were counterbalanced across subjects.

### Experimental task

Trials were signaled by the illumination of the nose-poke light (Med Associates, St Albans, VT) inside the box. When the light was on, a nose-poke into the lighted opening resulted in the nose-poke light turning off and four retractable levers (Med Associates, St Albans, VT) extending on the opposite wall. A press of one of the four levers initiated (a) the delivery of a food pellet according to the flavor assigned to that lever as well as (b) the retraction of all levers. After a pellet was delivered, the nose-poke light was turned on again for the next trial. During the experiment, the spontaneous choices and corresponding response times were recorded (see Text S1 for details). All experimental events were coordinated using MED-PC software (Med Associates, St Albans, VT).

### Estimation of parameters in the inter-choice interval (ICI) distribution

We estimated the value of 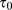 as the crossover point from the power-law to Weibull distribution, which would be represented as the local minimum value between these two distributions. Thus we calculated the value of 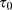 for individual rats as the local minimum of the probability density function of ICIs in the range between 50 and 1000 seconds. For short ICIs, we estimated the power-law exponent 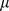 based on maximum likelihood estimation and selected the minimum time boundary 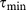, which provides the minimum value of the Kolmogorov-Smirnov goodness-of-fit statistic D [95]. For longer ICIs, the scale and shape parameters 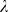 and 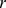 for the Weibull distribution were estimated by using a Matlab function, wblfit.m, on the basis of maximum likelihood estimation.

The parameters of the dual-state model were estimated from the empirical data for individual rats. We assumed that the ICIs in the active states would be smaller than the periods of inactive states. For simulation, we set the time constant 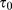, the power-law exponent 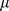 and the shape parameters for the light and dark cycles, 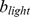 and 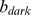, as free parameters. Activities of empirical data were grouped into an active state when their ICIs were less than 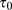, and separated into independent active states if the ICI was larger than 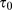; and thus the inactive state was defined as the time between the last event in a given active state and the first event in the next active state. Once the active and inactive states were determined, we estimated the average rate of the inactive period 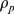, i.e., the reciprocal of the mean inactive duration, the probability of beginning an active state during a particular hour of the day 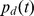, and the transition rate 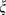 from the empirical data. Using these parameters, we generated simulated time series with the dual-state model. We estimated free parameters, 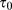, 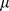, 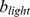, and 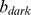, for each rat by using a least-area estimation [6], which provides the best-estimated parameters that minimize the area test static between the cumulative ICI distributions of the empirical and simulated data in a log-log scale.

### Model comparisons

To compare the fit of the dual-choice model with that of its nested models, i.e. the goal-directed or habit choice models alone, we used likelihood ratio tests and the Bayesian information criterion (BIC) [73] as follows:

BIC = −2•LL + k•ln N

where LL is the log-likehood of the model, k is the number of parameters of the model, and N is the number of trials. To examine how much better the models fit to empirical data compared to a random choice model, we calculated a pseudo-*r* ^2^ statistic defined as (R-L)/R, where R is the log-likelihood of the random choice model and L is that of our models [88]. A higher value indicates a better model fit.

## Supporting Information

Figure S1 Illustration of the experimental apparatus. The rat was required to nose-poke and then press one of four levers to receive the particular flavored food pellet in the corresponding receptacle. Water was freely accessible and located above the nose-poke hall.
(TIF)

Figure S2 Comparison of choice models for an example rat (subject 7). (A) The cumulative run distributions of the empirical data and the model predictions. (B) The cumulative choice frequency of the empirical data for all four ranks (C–G) The prediction of the dual-control model (Dual); the Goal_c+u_ model; the Goal_c_ model; the Habit model; and the Goal_c_+Habit model for all four ranks. (B–G) Red, orange, green, and blue represent the rank order from rank 1 to rank 4, respectively.
(TIF)

Figure S3 Comparisons of the simulation of the dual-state model with the empirical data. Cumulative ICI distributions of the empirical data (black squares) and the simulated data (red circles) are presented in a log-log scale for all 12 rats.
(TIF)

Figure S4 Comparisons of a choice sequence generated from the dual-control model with the empirical data. Cumulative run distributions of the empirical data and the simulated data are displayed in a log-log scale for all 12 rats. The black squares denote the empirical data and the blue circles the simulated data. In addition, cumulative choice frequency graphs for each rank for both the empirical data (solid lines) and simulation (dashed lines) are displayed. Red, orange, green, and blue represent the rank order from rank 1 to rank 4, respectively.
(TIF)

Table S1 The estimated parameters from 12 subjects for choice models. Values are given as mean (s.e.m).
(DOCX)

Text S1 Supporting text. Experiment apparatus, data pre-processing, choice model comparison, and appendix for modeling results of all subjects are described.
(DOCX)
