# Supplementary figures and images for: Bursts and Heavy Tails in Temporal and Sequential Dynamics of Foraging Decisions
Source: PLoS Comput Biol. 2014 Aug 14;10(8):e1003759. doi: 10.1371/journal.pcbi.1003759 (PMC4133158; doi:10.1371/journal.pcbi.1003759)

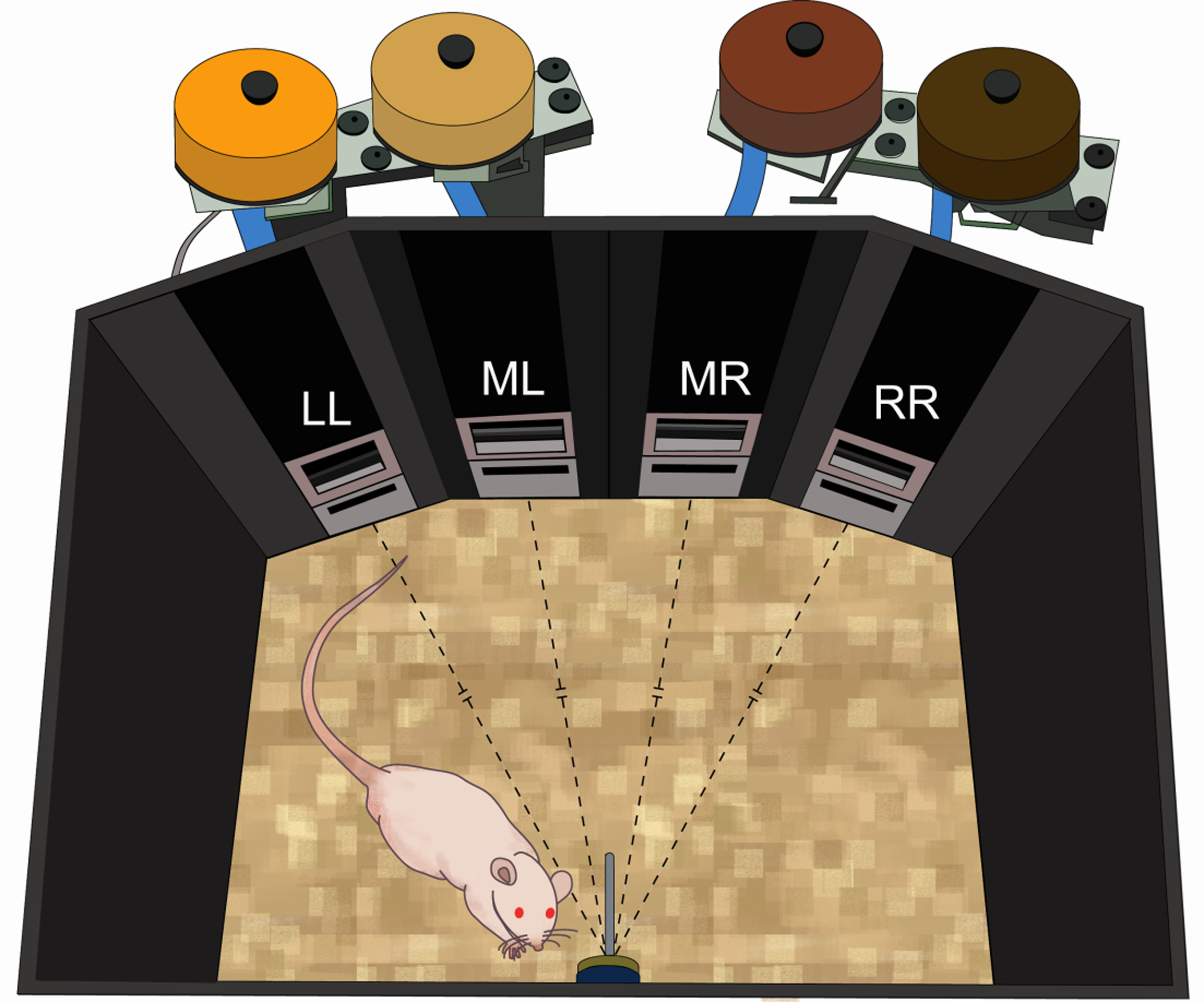

Supplement: Figure S1 — Illustration of the experimental apparatus. The rat was required to nose-poke and then press one of four levers to receive the particular flavored food pellet in the corresponding receptacle. Water was freely accessible and located above the nose-poke hall. (TIF) [file pcbi.1003759.s001.tif]

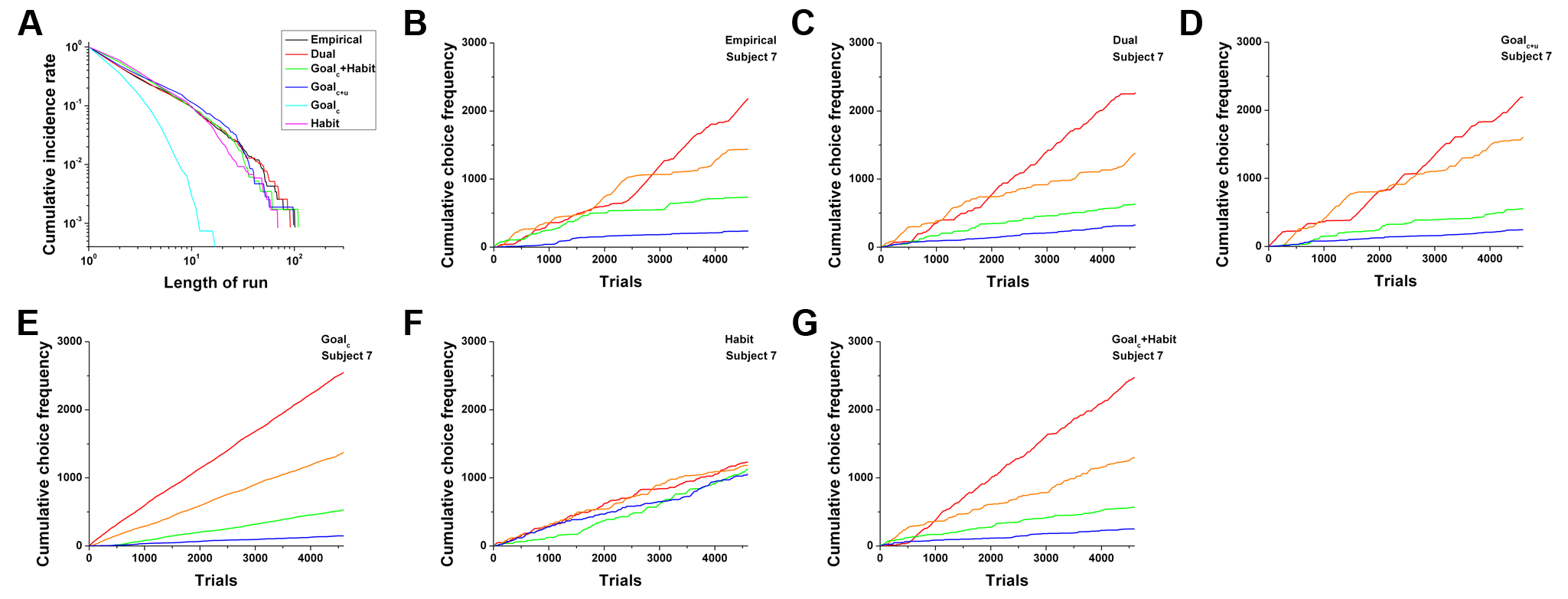

Supplement: Figure S2 — Comparison of choice models for an example rat (subject 7). (A) The cumulative run distributions of the empirical data and the model predictions. (B) The cumulative choice frequency of the empirical data for all four ranks (C–G) The prediction of the dual-control model (Dual); the Goalc+u model; the Goalc model; the Habit model; and the Goalc+Habit model for all four ranks. (B–G) Red, orange, green, and blue represent the rank order from rank 1 to rank 4, respectively. (TIF) [file pcbi.1003759.s002.tif]

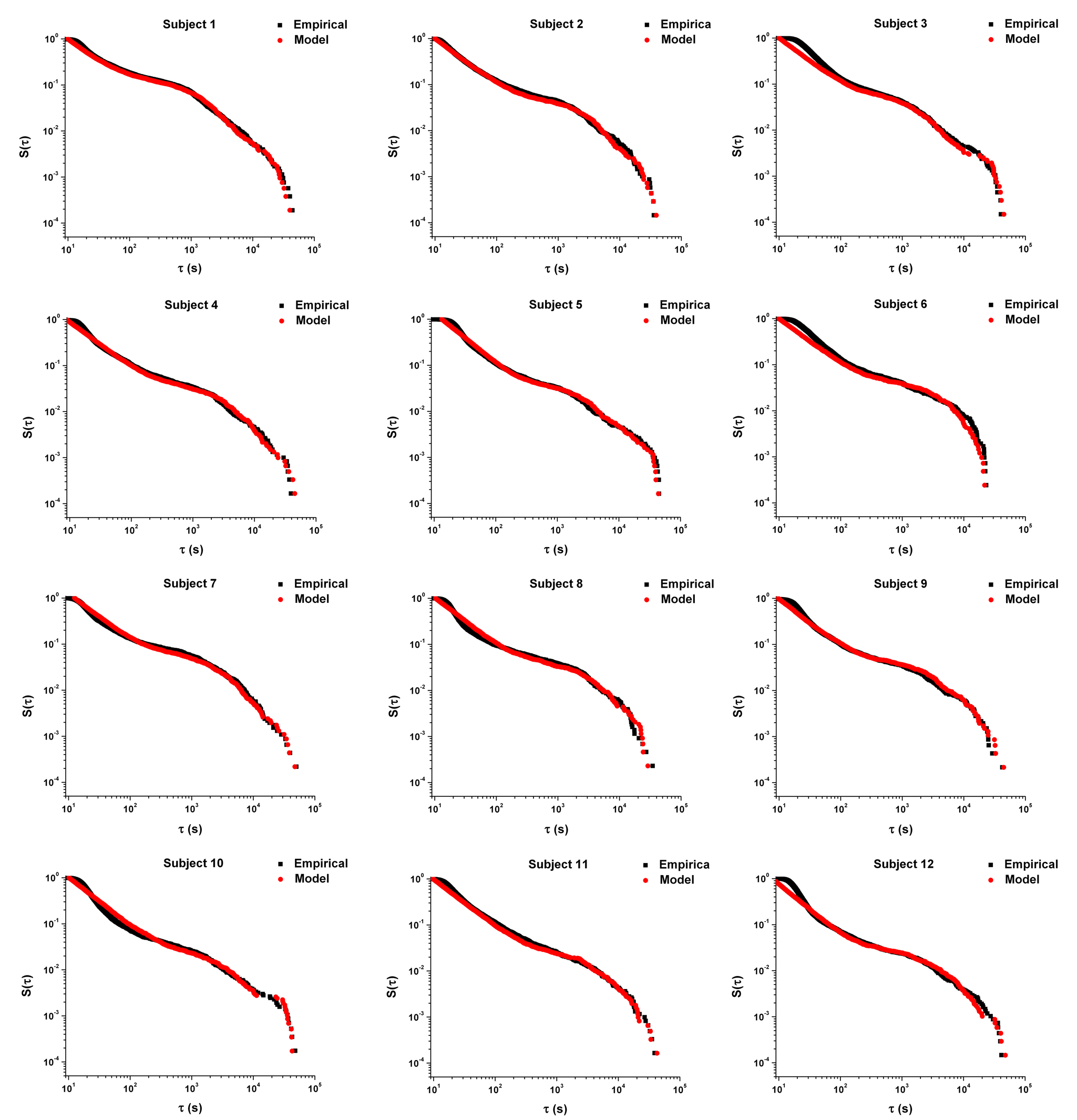

Supplement: Figure S3 — Comparisons of the simulation of the dual-state model with the empirical data. Cumulative ICI distributions of the empirical data (black squares) and the simulated data (red circles) are presented in a log-log scale for all 12 rats. (TIF) [file pcbi.1003759.s003.tif]

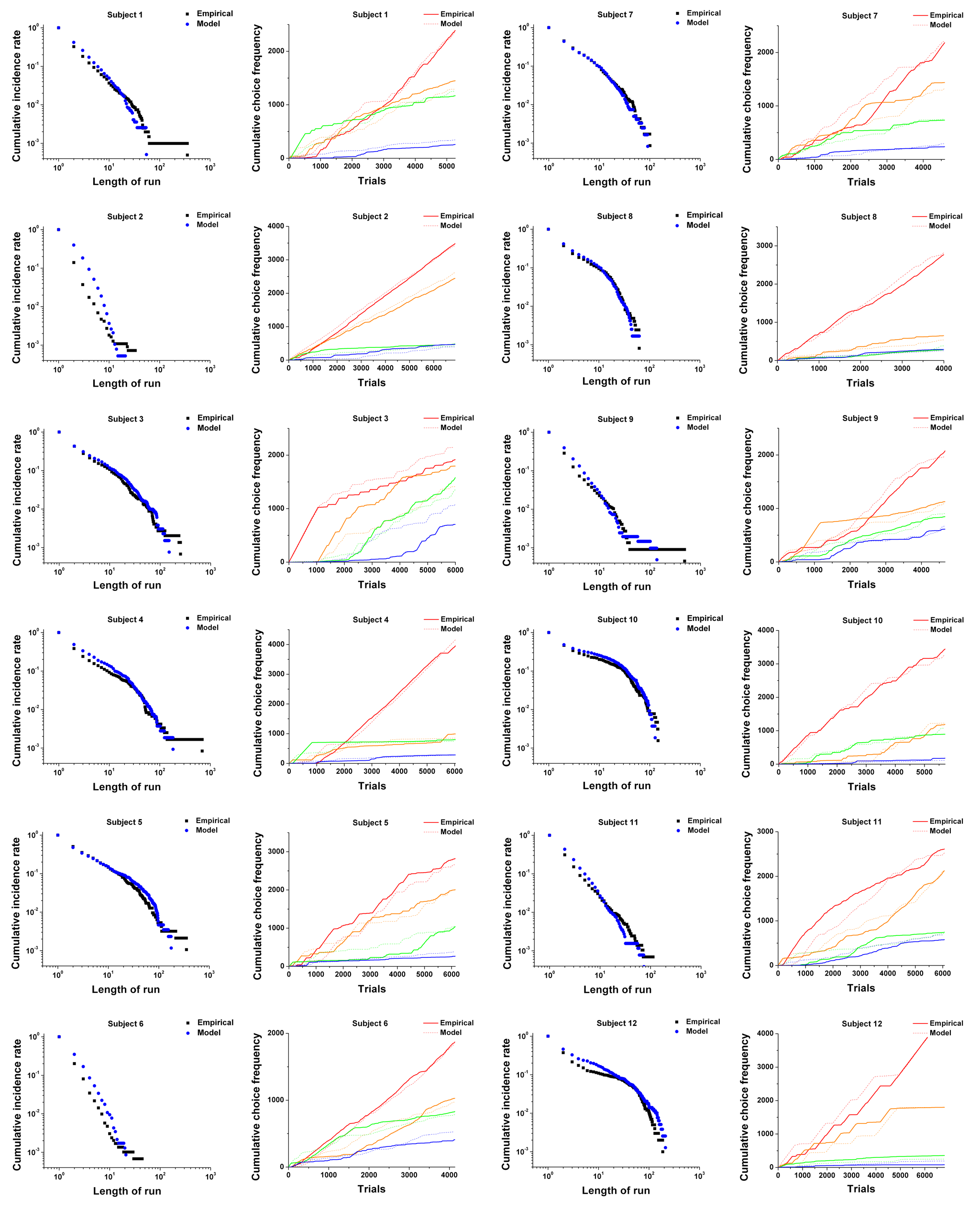

Supplement: Figure S4 — Comparisons of a choice sequence generated from the dual-control model with the empirical data. Cumulative run distributions of the empirical data and the simulated data are displayed in a log-log scale for all 12 rats. The black squares denote the empirical data and the blue circles the simulated data. In addition, cumulative choice frequency graphs for each rank for both the empirical data (solid lines) and simulation (dashed lines) are displayed. Red, orange, green, and blue represent the rank order from rank 1 to rank 4, respectively. (TIF) [file pcbi.1003759.s004.tif]
