# Supplementary material for: Bursts and Heavy Tails in Temporal and Sequential Dynamics of Foraging Decisions
Source: PLoS Comput Biol. 2014 Aug 14;10(8):e1003759. doi: 10.1371/journal.pcbi.1003759 (PMC4133158; doi:10.1371/journal.pcbi.1003759)
Supplement: Table S1 — The estimated parameters from 12 subjects for choice models. Values are given as mean (s.e.m). (DOCX) [file pcbi.1003759.s005.docx]

| Table S1  *Parameter estimates of choice models* | | | | | |
| --- | --- | --- | --- | --- | --- |
|  | Dual | Goal_c+u_ | Goal_c_ | Habit | Goal_c_+Habit |
| $s$ | 8.08 ± 1.14 | 10.69 ± 1.17 | 3.62 ± 0.49 | - | 2.59 ± 0.45 |
| $\alpha_{c}$ | 0.27 ± 0.03 | 0.39 ± 0.04 | 0.40 ± 0.12 | - | 0.21 ± 0.08 |
| $\alpha_{u}$ | 0.16 ± 0.04 | 0.11 ± 0.02 | - | - | - |
| $\beta_{G}$ | 3.57 ± 0.16 | 4.30 ± 0.12 | 6.95 ± 0.55 | - | 2.50 ± 0.12 |
| $\beta_{H}$ | 1.34 ± 0.18 | - | - | 3.75 ± 0.11 | 3.26 ± 0.14 |
| $\omega$ | 73.4 ± 22.9 | - | - | 11.63 ± 2.06 | 0.39 ± 0.04 |
