# Supplementary material for: Bursts and Heavy Tails in Temporal and Sequential Dynamics of Foraging Decisions
Source: PLoS Comput Biol. 2014 Aug 14;10(8):e1003759. doi: 10.1371/journal.pcbi.1003759 (PMC4133158; doi:10.1371/journal.pcbi.1003759)
Supplement: Text S1 — Supporting text. Experiment apparatus, data pre-processing, choice model comparison, and appendix for modeling results of all subjects are described. (DOCX) [file pcbi.1003759.s006.docx]

**Supporting Information**

**Bursts and Heavy Tails in Temporal and Sequential Dynamics of Foraging Decisions**

**Kanghoon Jung^a,b^, Hyeran Jang^a^, Jerald D. Kralik^b^, Jaeseung Jeong^a,^***

^a^Department of Bio and Brain Engineering,

Korea Advanced Institute of Science and Technology (KAIST),

Daejeon 305-701, Korea

^b^Department of Psychological and Brain Sciences,

Dartmouth College, Hanover, NH 03755, USA

**Experiment apparatus**

The experiment used operant chambers (60 cm wide × 40cm deep × 40 cm high) made from black plastic board, placed in a room with controlled temperature and a 12 hour dark/ light cycle (Figure S1). All subjects were housed individually in an operant task chamber for two weeks with hard wood bedding (Beta chip) on the cage floor. In each chamber, a light-equipped nose-poke panel (ENV-114M; Med Associates, St Albans, VT) was installed at the center of the front wall, below the water container. On the opposite wall, four retractable levers (ENV-112CM; Med Associates, St Albans, VT) were mounted below each food receptacle (ENV-200R2M; Med Associates, St Albans, VT), which were connected to each of the four pellet dispensers (ENV-203; Med Associates, St Albans, VT). The four levers were assigned as left most (LL), middle left (ML), middle right (MR) and right most (RR). The distances between the nose poke and each lever were the same for all four levers to exclude a behavioral bias due to distance, and thus the wall with each lever was bent at an angle (see Figure S1).

**Data pre-processing**

The animals were required to learn the task themselves without operant behavior training. The response time was defined as the latency from a nose-poke to a lever press. Trials with response times less than five seconds were considered successful. It was assumed that a rat learned the behavioral paradigm once the percentage of successful trials exceeded 90% over the last 20 trials. Trials after learning were used for data analysis. The average median response time across all subjects was 3.70 ± 0.34 (averaged median ± s.e.m.) seconds after learning the task.

**Choice model comparison**

To determine which choice models provided a better fit to the empirical choice patterns, we simulated choice sequences based on the dual-control model as well as models based on the nested components of the dual control model: (1) The goal-directed control model alone with update for the chosen option and decay for unchosen options (Goal_c+u_); (2) the goal-directed control model alone with update for the chosen option only, i.e., standard TD learning for the chosen option (Goal_c_); (3) the habitual control model alone (Habit); (4) the submodel of dual-control (Goal_c_+Habit) composed of the mixture of Goal_c_ (with update for the chosen option only) and Habit; and (5) The dual-control model (Dual) composed of the mixture of Goal_c+u_ (with update for the chosen option and decay for the unchosen options) and Habit.

We compared the run distribution and choice frequency resulting from the simulation of each model with the empirical data. We estimated the free parameters of the models for each subject by minimizing the negative log-likelihood of the empirical choice sequence across individuals (Table S1) [1]. We present the predictions of the choice models with the best-estimated parameters for a representative rat in Figure S2. To assess which model could best capture the underlying processes that determine the sequential dynamics, we compared the run distribution and the cumulative choice frequency of each model. The Goal_c_, in which the decay for unchosen options was not included, did not account for the heavy-tailed run distribution of the empirical data, instead exhibiting a rapid decay. This suggests that the update for the chosen option alone based on the reward prediction error is not sufficient to account for the persistent component of choice behavior, which generates very long runs. In contrast, the other models captured the heavy-tail property well. In addition, the Habit model did not precisely capture the degree of bias among the options with respect to rank order in the cumulative choice frequency distributions. Furthermore, given dynamic changes in preference across trials, the Dual model exhibited a closer prediction to the empirical data than the Goal_c+u_ and Goal_c_+Habit models, and produced the highest likelihood of the model among all models. Taken together, the Dual model provided the most accurate descriptions of the sequential dynamics with respect to choice persistence, choice bias among ranks, dynamics across trials, and the likelihood of the model.

**Appendix**

Modeling comparisons for the dual-state and dual-control models with the empirical data for each of the 12 subjects. Parameters are estimated for each subject (See *Estimation of parameters in the inter-choice interval (ICI) distribution* in *Methods* for the dual-state model and *Choice model comparison* in *Text S1)*. Figure S3 shows comparisons of the simulation of the dual-state model with the empirical data. Cumulative ICI distributions of the empirical data (black squares) and the simulated data (red circles) are presented in a log-log scale for all 12 rats. Figure S4 shows comparisons of a choice sequence generated from the dual-control model with the empirical data. Cumulative run distributions of the empirical data and the simulated data are displayed in a log-log scale for all 12 rats. The black squares denote the empirical data and the blue circles the simulated data. In addition, cumulative choice frequency graphs for each rank for both the empirical data (solid lines) and simulation (dashed lines) are displayed. Red, orange, green, and blue represent the rank order from rank 1 to rank 4, respectively.

**References**

1. Daw ND (2011) Trial-by-trial data analysis using computational models. In: Delgado MR, Phelps EA, Robbins TW, editors. Decision Making, Affect, and Learning: Attention and Performance XXIII. New York: Oxford Universtiy Press.
